# The quality and reliability of YouTube video content about gingival recession on different time periods

**DOI:** 10.7717/peerj.19653

**Published:** 2025-07-07

**Authors:** Beyza Bozoklu, Nülüfer Demir, Merve Akbaş, Hatice Sena Öner, Duygu Yaman

**Affiliations:** 1 Department of Periodontology, Istanbul University, Istanbul, Turkey; 2 Institute of Graduate Studies in Health Sciences, Istanbul University, Istanbul, Turkey

**Keywords:** Gingival recession, COVID-19, YouTube, Gum recession

## Abstract

**Background:**

Gingival recession is a mucogingival problem that can cause esthetic concerns and sensitivity. While some patients remain unaware of it, others seek solutions through various means. The COVID-19 pandemic has increased the role of social media in accessing health information. The frequent use of YouTube has prompted researchers to evaluate its contents quality and reliability. This study aims to examine this issue by comparing two periods: before and after COVID-19.

**Methods:**

The first 100 videos for the keyword ‘receding gums’ shared in 2019 and 2024 were analyzed. A total of 23 and 35 videos were included, respectively. Quality was evaluated using Video Quality Index (VIQI) and Global Quality Scale (GQS), reliability with DISCERN and Modified DISCERN and content through an approach developed by the authors. Videos were also assessed based on their characteristics and sources. Data were analyzed using IBM SPSS 26.0. For two-group comparisons, independent sample *t*-tests were applied to normally distributed data, while the Mann–Whitney *U* test was used for non-normally distributed data. Categorical variables were analyzed with the Chi-square test. Correlations between variables were examined using Spearman’s correlation analysis.

**Results:**

According to content analysis, useful videos were found to have higher scores of quality and reliability indices in both the 2019 and 2024 groups (*p* < 0.001). In 2024, the amount of videos uploaded by dental professionals was significantly more ‘useful’ compared to those from other sources (*p* = 0.45). There was a tendency of increase in sharing videos by healthcare professionals following the COVID-19 pandemic.

## Introduction

Gingival recession is defined as the apical migration of the gingival margin, resulting in attachment loss and exposure of the root surface (Jepsen et al., 2018). This condition can lead to clinical manifestations such as dentin sensitivity, aesthetic concerns, a tendency for root caries development, and ultimately progression to tooth loss (Pradeep et al., 2012). Gingival recession is observed in patient groups with both good and poor oral hygiene habits (Loe, Anerud & Boysen, 1992). While some patients are unaware of the presence of gingival recession, others complain of sensitivity and/or aesthetic issues (Wennstrom, 1996). A study on patients’ perceptions of gingival recession found that patients were more aware of recession in the anterior region and of deeper recessions. Younger and female individuals were observed to be more aware compared to older and male individuals. Additionally, younger patients were more aware of sensitivity issues related to gingival recession and showed greater interest in seeking solutions to address their aesthetic concerns (Nieri et al., 2013).

Social media, with its accessibility and emergence as a primary source of information in recent times, has rapidly become an integral part of human life. People are spending more and more time on social media and the internet, with an increasing tendency to seek information online about various aspects of their lives (Song, Jung & Kim, 2016). It is undeniable that the recent COVID pandemic, which introduced the concept of staying and living at home, has significantly contributed to this trend (Brailovskaia, Margraf & Schneider, 2021; Liu et al., 2021). Social media is now widely recognized as a major source of health information, surpassing traditional media in reach and influence (Swire-Thompson & Lazer, 2020; Ghalavand & Nabiolahi, 2024). In fact, according to a study, people tend to seek assistance from social media before consulting a healthcare professional (Haslam et al., 2019).

While the development with social media greatly simplifies daily life, the characteristics of the information accessed add complexity to the situation. It has been noted that, on social media, misinformation spreads alongside accurate information, with one study indicating that misinformation spreads at a faster rate (Vosoughi, Roy & Aral, 2018). YouTube, founded in 2005, is one of the widely used platforms for sharing videos. However, with minimal restrictions on who can upload content, anyone can post videos, regardless of their qualifications. The changing lifestyles of spending more time at home due to the health conditions experienced all over the world in the last 5–6 years have pushed society to change their behavior to meet their needs through digital methods, which has accelerated the impact of social media on society. The need to obtain information is one of the areas where this change is most experienced. The ease with which people access misinformation, often even more readily than accurate information, poses a greater challenge in the healthcare sector compared to other fields. As a result, the characteristics of information shared in this domain have become a significant concern for healthcare authorities. This lack of regulation has led health professionals to study the quality and reliability of health-related videos available on the platform. Some of the studies conducted on this subject mention that the quality and reliability of the videos shared on social media are insufficient (Aksoy & Topsakal, 2022; Erturk-Avunduk & Delikan, 2023). It has been reported that the quality of the videos tends to decrease between two evaluations made over a period of 1 year (Hamdan et al., 2019).

In light of this information, the aim of this study is to compare the YouTube videos shared between 2019–2024 in terms of quality, reliability and content and to evaluate the effect of society’s increased habit of obtaining information digitally on the information shared.

## Materials & Methods

### Study design and data collection

**Figure 1:**
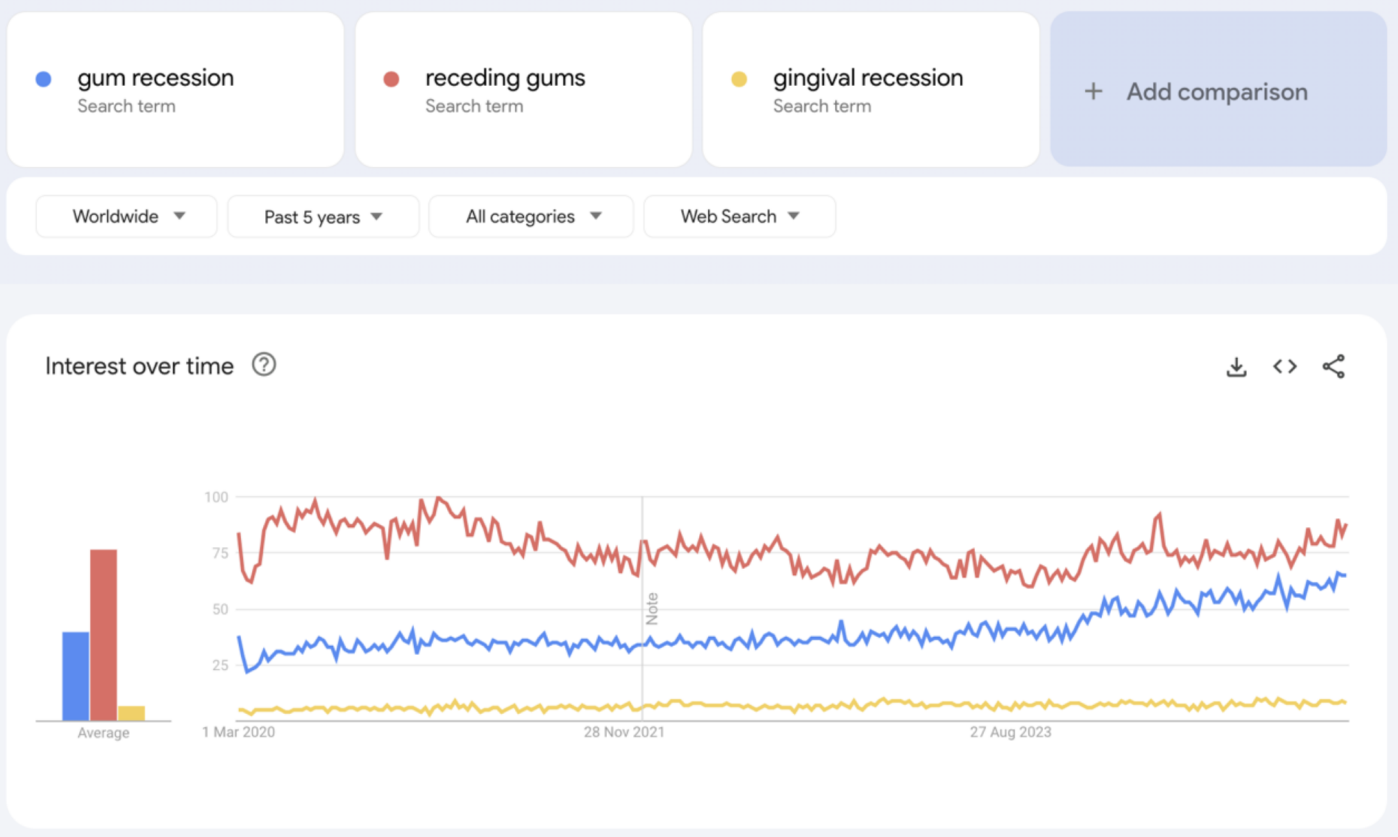
Comparison of possible keywords in Google Trends.

Since publicly accessible data was used, ethical committee approval was not required. Google Trends (Google Trends, 2021, Alphabet, USA) (https://trends.google.com) is an online platform aimed at identifying the frequency of term searches over a specific time period. Using incognito mode, the terms ‘receding gums,’ ‘gingival recession,’ and ‘gum recession’ related to gingival recession were compared with the filters “worldwide” and “last 5 years.” It was determined that “receding gums” was the most searched term among these, and it was chosen as the keyword in the present study (Fig. 1). Searches were conducted on Google Videos, on 17 November 2024 by two experienced periodontists. The results were filtered by selecting only the YouTube page and the dates 01.01.2019–17.11.2019 and 01.01.2024–17.11.2024. The first 100 videos were assessed in our study. The video URLs were saved to access the same videos in subsequent searches. Videos were included if they were publicly accessible on YouTube, related to the selected keyword (“receding gums”), presented in English, had sufficient audio-visual clarity for evaluation, and were intended for a general (non-specialist) audience.

 Non-English videos were excluded based on language criteria. Videos targeted at specific professional audiences, such as conference or congress content, were excluded. Additionally, irrelevant, entertainment-based, commercial, and duplicated videos were removed from the analysis.

### Assessment of the videos

**Figure 2:**
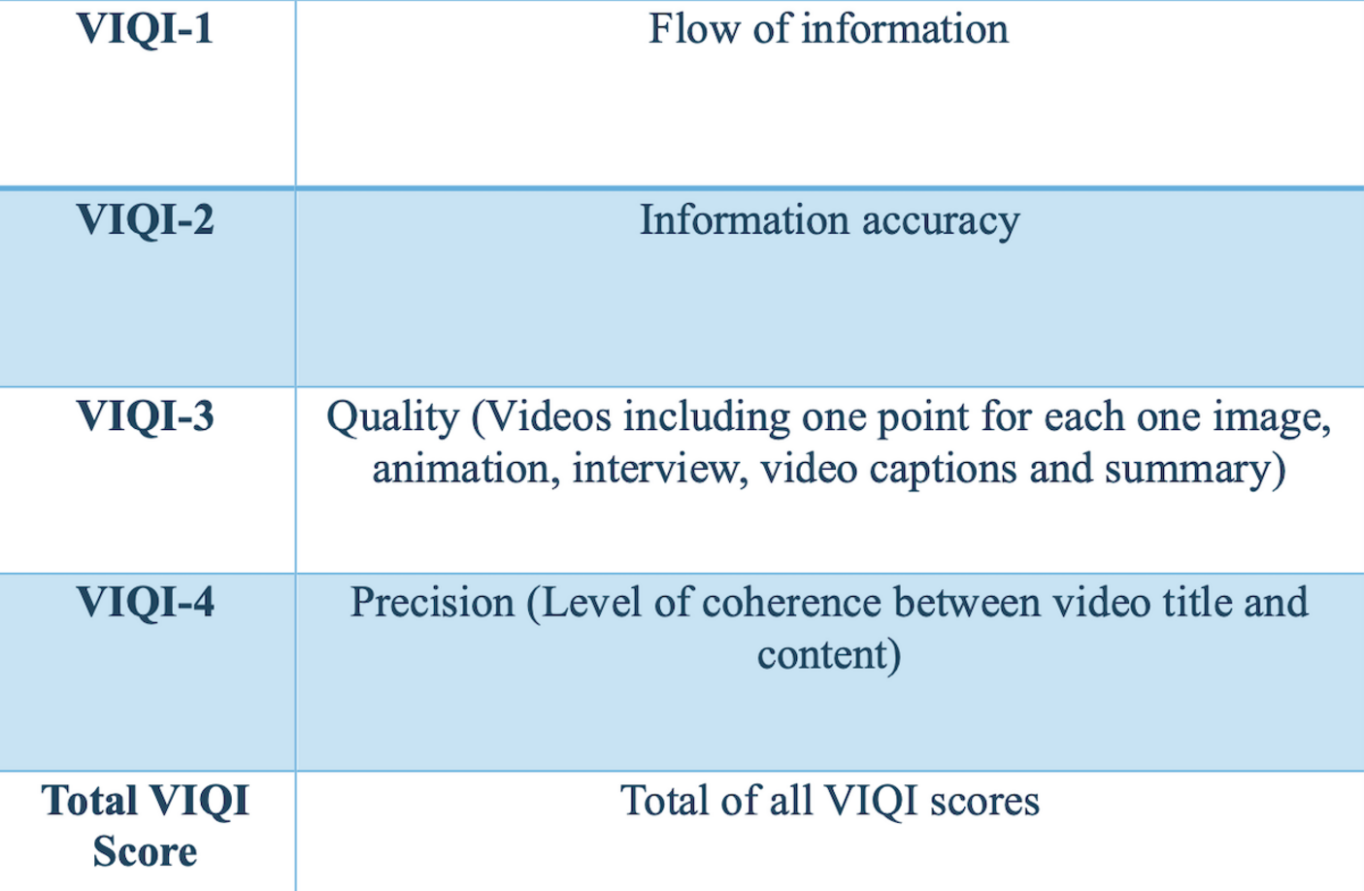
Video Quality Index (VIQI).

**Figure 3:**
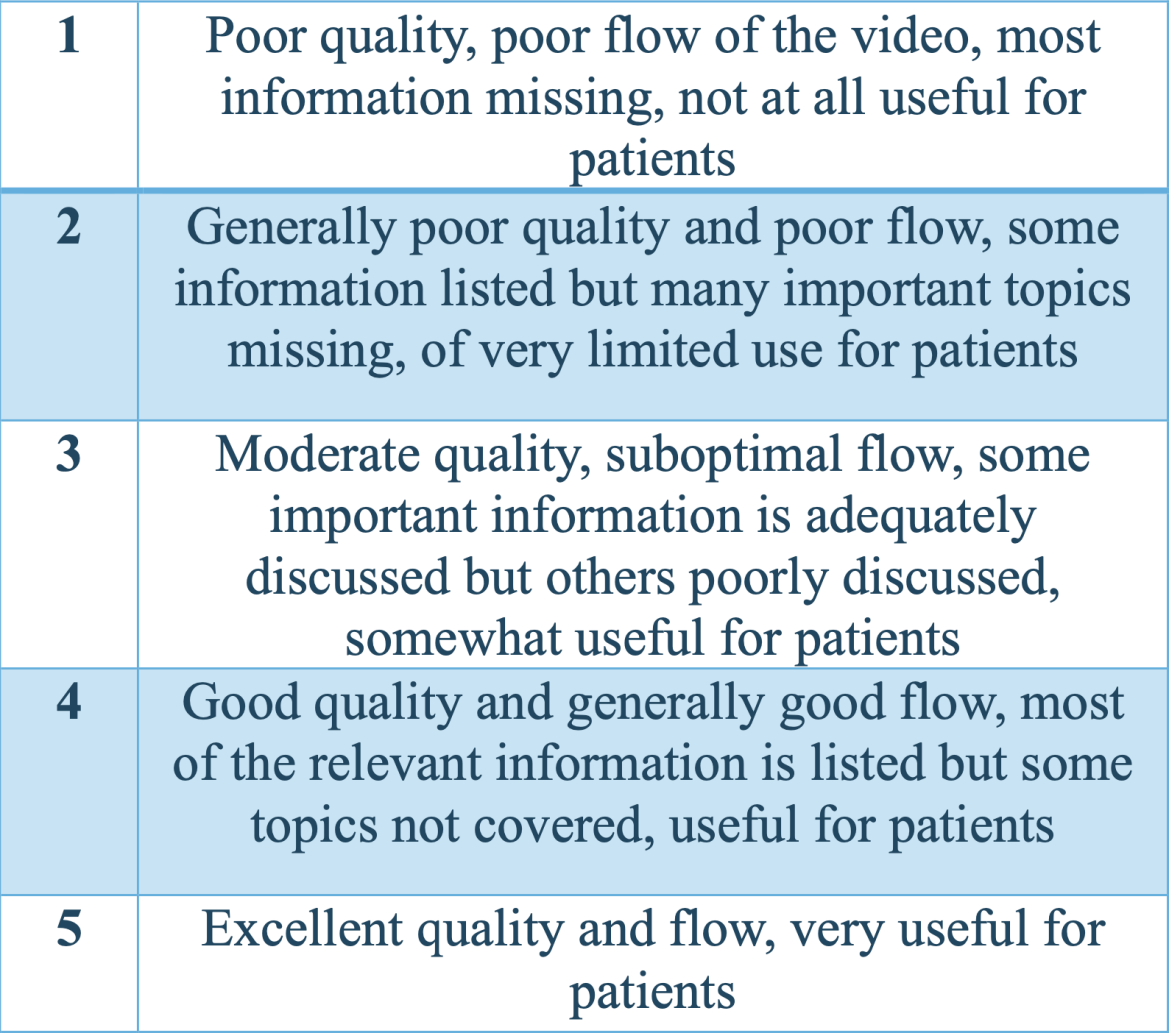
Global Quality Scale (GQS).

**Figure 4:**
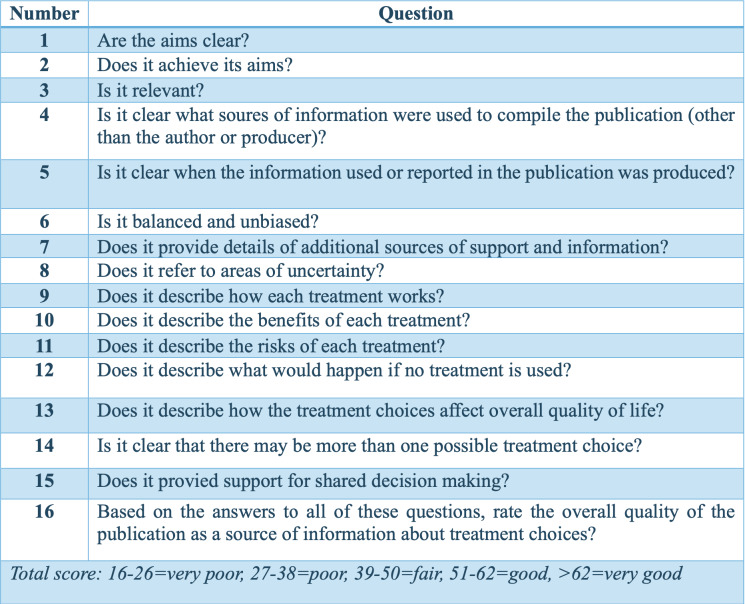
DISCERN.

**Figure 5:**
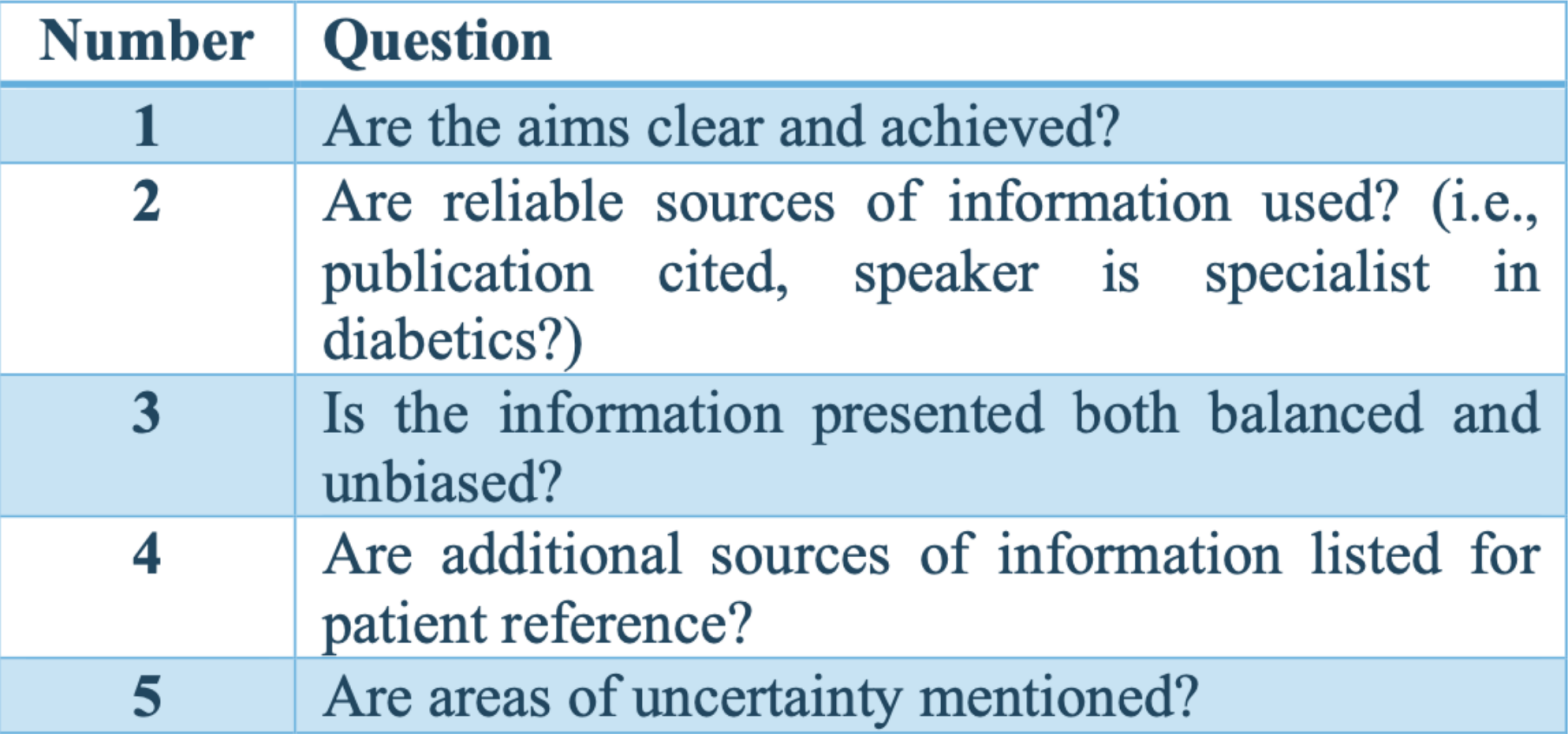
Modified DISCERN.

**Figure 6:**
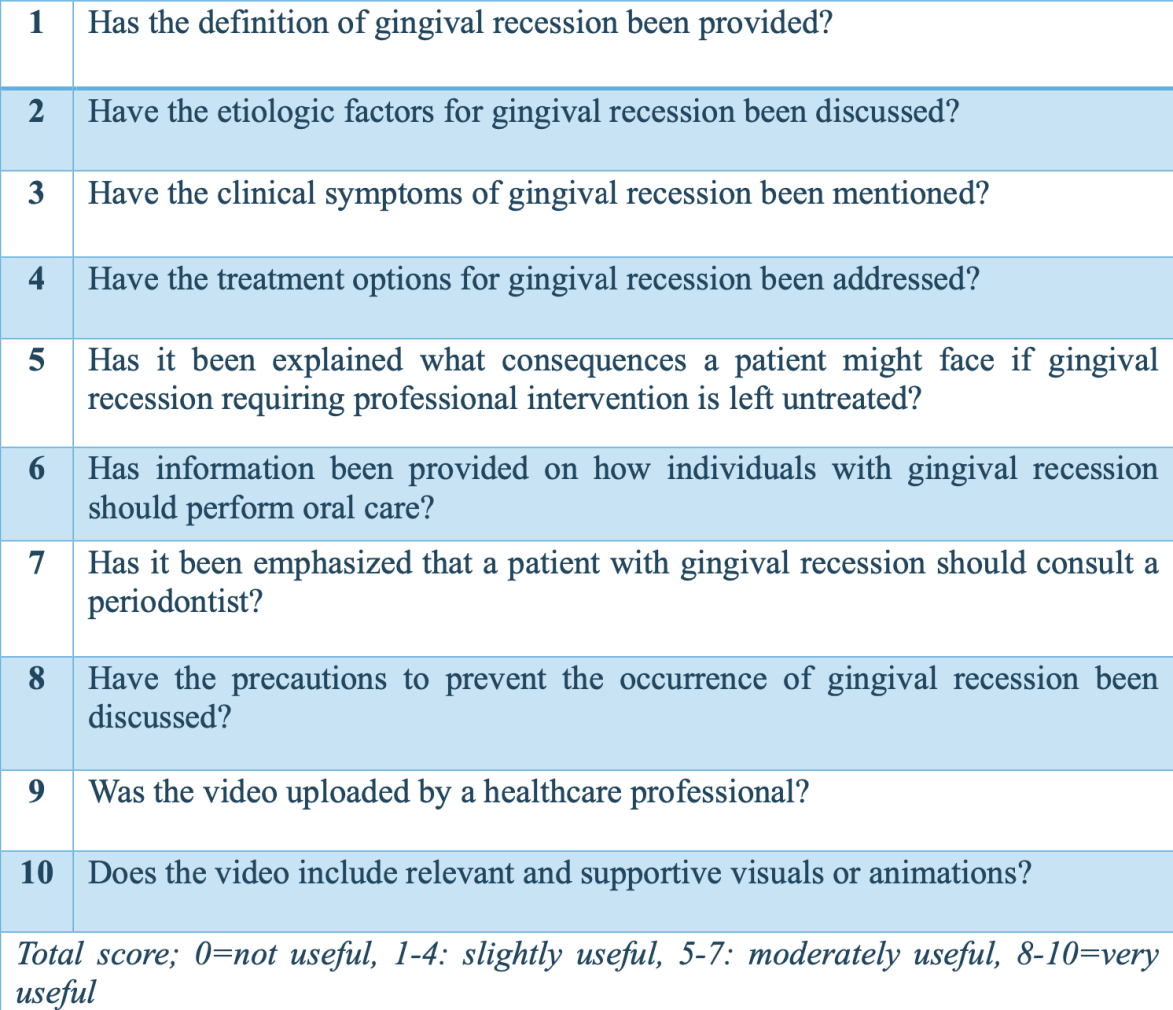
Content analysis (If yes, score 1; if no, score:0).

Regarding the evaluation of the videos, Video Quality Index (VIQI) was used to assess overall video quality, focusing on content accuracy, source credibility, and production quality; DISCERN (Charnock et al., 1999) to evaluate the reliability and clarity of the health information; the Modified DISCERN index to refine the depth and comprehensiveness of the content; and the Global Quality Scale (GQS) (Bernard et al., 2007) to evaluate the overall quality and reliability, considering both content and presentation (Figs. 2–6). Additionally, a content analysis index customized by the authors was used, similar to previous studies (Ince Kuka & Gursoy, 2024; Gezer et al., 2025). Video characteristics were assessed by recording details such as the video’s duration, the number of likes, comments, and its source.

### Statistical analysis

Data were analyzed using IBM SPSS 26.0 with a significance level of 95% (*p* = 0.05). Descriptive statistics are given as mean, standard deviation, median, IQR (%75–%25 percentile) and percentage and the distribution of normality was checked using the Shapiro–Wilk test. In two-group comparisons the independent sample *t*-test was used for normally distributed data, whereas the Mann–Whitney *U* test was used for non-normally distributed data. Categorical comparisons were analyzed using the Chi-square test. Correlation between the variables was analyzed with Spearman correlation analysis. The inter-observer reliability was assessed using the Intraclass Correlation Coefficient (ICC).

## Results

**Figure 7:**
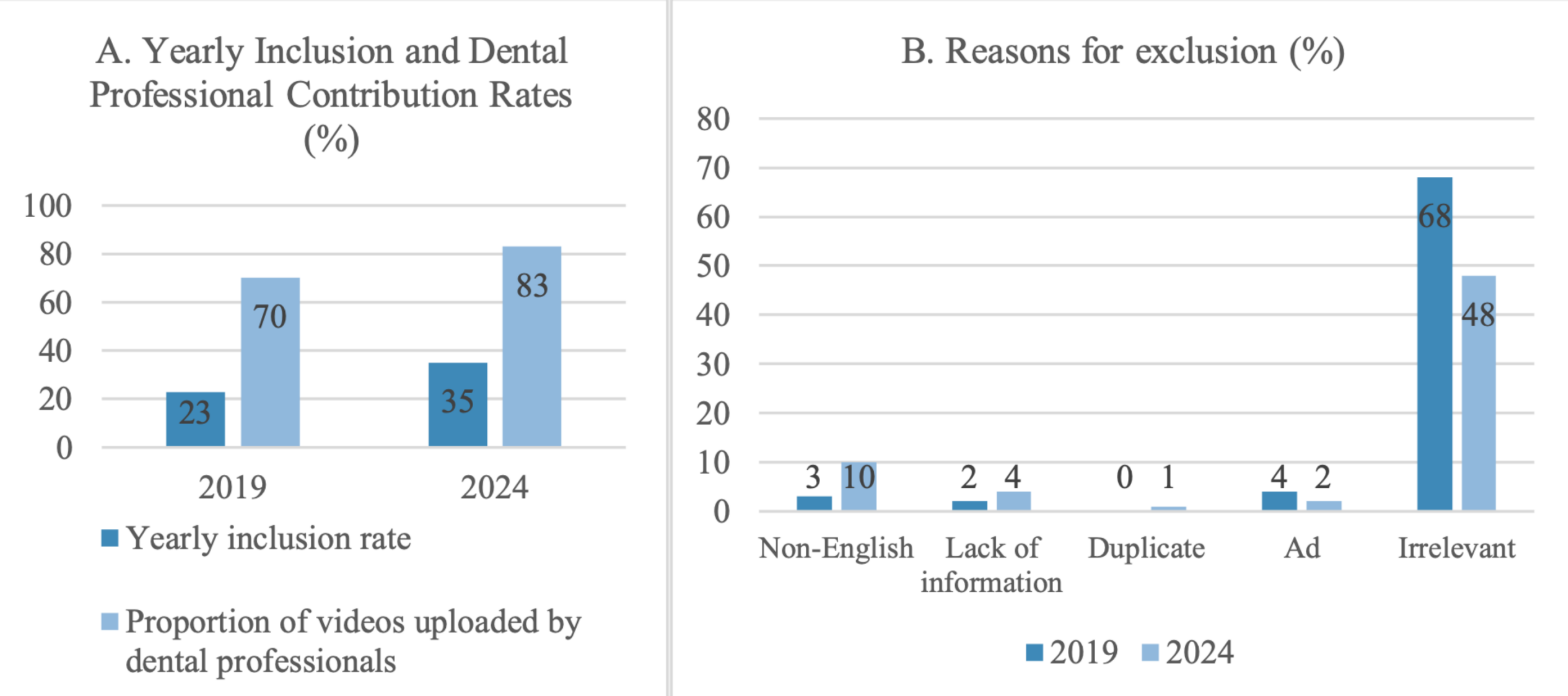
Overview of YouTube video analysis for 2019 and 2024. (A) Percentage of videos included in the study out of the initial 100 screened, and percentage of included videos uploaded by dentists, (B) Reasons for video exclusion presented as percentage of total screened videos.

A total of 100 videos meeting the established criteria were considered, with 23 videos from the 2019 group and 35 from the 2024 group included in the study. In 2019, 70% of the videos were uploaded by dentists, compared to 83% in 2024. Among the 100 videos from 2019, 3% were non-English, 2% lacked information, 4% were product advertisements, and 68% were irrelevant; whereas the 100 videos from 2024, 10% were non-English, 4% lacked any information, 1% were duplicates, 2% were product advertisements, and 48% were irrelevant to the topic (Fig. 7).

A high level of agreement was observed between the two periodontists, with an ICC greater than 90%. Therefore, the data were analyzed by calculating the inter-examiner mean value (Table 1). Table 2 shows the distribution of videos in each of the evaluated indices in different years. GQS revealed 52.2% of videos posted in 2019 and 37.2% of videos posted in 2024 were high quality. According to DISCERN scores, no videos posted in 2019 and 11.4% of videos posted in 2024 were very good. Content analysis revealed 17.4% of 2019 videos and 40% of 2024 videos were very useful.

**Table 1:** Inter-observer aggreement levels.

	**2019**			**2024**		
	1.	2.	ICC	1.	2.	ICC
GQS (1–5)	3 ± 1	3 ± 1	0.892	3 ± 1	3 ± 1	0.981
VIQI	13 ± 5	13 ± 6	0.991	12 ± 5	13 ± 5	0.993
DISCERN	37 ± 12	39 ± 14	0.986	39 ± 17	40 ± 17	0.994
Modifiye DISCERN	13 ± 5	14 ± 6	0.930	14 ± 6	15 ± 6	0.989
Content analysis	5 ± 3	5 ± 3	0.989	6 ± 3	6 ± 3	0.988

**Notes.**

GQS Global quality scale VIQI Video information and quality index ICC Intraclass correlation coefficient

**Table 2:** Distribution of the videos according to indices and years.

**Indices**	**Values**	**2019**		**2024**	
		*n*	%	*n*	%
DISCERN	Very poor (16–26)	5	21.7	11	31.4
	Poor (27–38)	8	34.8	9	25.7
	Fair (39–50)	6	26.1	4	11.4
	Good (51–62)	4	17.4	7	20
	Very good (>62)	0	0	4	11.4
GQS	Low quality (1 or 2)	9	39.1	16	45.7
	Medium (3)	2	8.7	6	17.1
	High quality (4 or 5)	12	52.2	13	37.2
CONTENT ANALYSIS	Not useful (0)	1	4.4	3	8.6
	Slightly useful (1–4)	9	39.1	9	25.7
	Moderately useful (5–7)	9	39.1	9	25.7
	Very useful (8–10)	4	17.4	14	40

**Notes.**

GQS, Global quality scale.

Table 3 shows descriptive characteristics and comparison of videos posted in 2019 and in 2024. The median values of duration time for videos posted in 2019 and 2024 were 3.7 and 6.3, respectively. The median values of likes and comments of the videos were slightly higher in 2024, while the median value of views was lower. None of these differences were significant (*p* > 0.05). Similarly, there were no significant differences between the videos posted in 2019 and in 2024 in terms of quality scores (GQS and VIQI), reliability scores (DISCERN and Modified DISCERN) and content analysis scores. 70% of videos in 2019 and 83% of videos in 2024 were posted by dental professionals, which did not differ significantly.

**Table 3:** Descriptive characteristics and comparison of 2019 and 2024 videos.

	**Year**		
	2019 (*n* = 23)	2024 (*n* = 35)	*p*
**Video characteristics**	median (IQR)		
*Duration time*	3.7 (8.7)	6.3 (10.9)	0.301
Views	3.982 (7,729)	1.549 (5,667)	0.199
Likes	21 (52)	29 (380)	0.813
Comments	3 (43)	4 (43)	0.971
**Quality**	
GQS	4 (2)	3 (2)	0.738
VIQI	14 (11)	13 (10)	0.560
**Content analysis**	5 (4)	6 (5)	0.081
**Reliability**	mean ± sd		
DISCERN	39 ± 14	40 ± 17	0.687
Modified DISCERN	14 ± 6	15 ± 6	0.629
**Source of the video**	*n* (%)		
Health professional	16 (%70)	29 (%83)	0.235
Other	7 (%30)	6 (%17)	

**Notes.**

sd standard deviation IQR %75–25 percentile GQS Global quality scale VIQI Video information and quality index

*p*, Mann–Whitney *U* test, values presented as median (IQR); *p*, Chi-square test, values presented as *n* (%); p*, Independent samples *t*-test, values presented as mean (standard deviation).

Table 4 reveals comparison of the videos posted in 2019 and in 2024 according to content analysis scores. In order to be more understandable statistically, content analysis was grouped into two categories, “not useful” including not useful and slightly useful scores, and “useful” including moderately useful and very useful scores. A total of 20 videos were classified as “not useful”, while 38 were classified as “useful”. Quality scores (GQS and VIQI) of “useful” videos were significantly higher in both videos posted in 2019 and 2024 (*p* < 0.001). As represented by mean values of DISCERN and Modified DISCERN scores, the reliability scores were also significantly higher in “useful” videos of both 2019 and 2024 videos (*p* < 0.001 and *p* < 0.05). Regarding the source of the videos, the amount of “useful” videos posted by dental professionals in 2024 were significantly higher than those posted by other sources (*p* = 0.45).

**Table 4:** Comparison of the video characteristics, indices and source according to the content analysis.

	**Content analysis**					
	2019			2024		
	Not useful (*n* = 11)	Useful (*n* = 12)	*p*	Not useful (*n* = 9)	Useful (*n* = 26)	*p*
**Video characteristics**	median (IQR)			median (IQR)		
Duration time	2.2 (3.3)	4.7 (7.8)	0.165	1 (8.6)	6.8 (8.9)	0.151
Views	3,434 (6,817)	5,604 (12,614)	0.242	495 (4,230)	1,592 (5,392)	0.408
Likes	12 (44)	24 (66)	0.181	6 (187)	31 (379)	0.473
Comments	2 (34)	3 (49)	0.512	0 (26)	8 (43)	
**Quality**	
GQS	2 (2)	4 (1)	***p* < 0.001**	2 (1)	4 (1)	***p* < 0.001**
VIQI	9 (7)	18 (4)	***p* < 0.001**	8 (3)	15 (7)	***p* < 0.001**
**Reliability**	mean ± sd			mean ± sd		
DISCERN	27 ± 9	49 ± 8	***p* < 0.001**	23 ± 6	46 ± 16	***p* < 0.001**
Modified DISCERN	11 ± 7	17 ± 4	**0.014**	9 ± 4	17 ± 5	***p* < 0.001**
**Source of the video**	*n* (%)			*n* (%)		
Health professional	6 (%55)	10 (%89)	0.296	5 (%56)	24 (%92)	**0.045**
Other	5 (%45)	2 (%11)		4 (%44)	2 (%8)	

**Notes.**

sd, standard deviation; IQR, %75–25 percentile, *p*, correlation coefficient.

Bold values indicate statistical significance.

*p*: Mann–Whitney *U* test, values presented as median (IQR); *p*: Chi-square test, values presented as *n* (%); *p*∗: Independent samples *t*-test, values presented as mean (standard deviation).

Table 5 reveals correlation between video characteristics and the indices including GQS, VIQI, DISCERN and modified DISCERN. In the 2019 group, GQS values were significantly positively correlated with like numbers (*p* = 0.033). Additionally, there was a negative significant correlation between the number of days since the upload of the videos and VIQI and DISCERN values (*p* = 0.030 and *p* = 0.031, respectively). Videos posted by dental professionals were significantly negatively correlated with DISCERN and Modified DISCERN scores (*p* = 0.015 and *p* < 0.001, respectively). Videos posted in 2024 were significantly correlated with reliability indices (*p* = 0.026 and *p* = 0.041, respectively). There was significant negative correlation between the videos uploaded by dental professionals and VIQI scores (*p* = 0.008). Regarding content analysis, “useful” videos exhibited significant positive correlation between all indices in both 2019 and 2024 groups (*p* < 0.001).

**Table 5:** Correlation between video characteristics and indices in different time periods.

	**2019** **(*n* = 23)**				**2024** **(*n* = 35)**				
Video characteristics	GQS	VIQI	DISCERN	Modified DISCERN	GQS	VIQI	DISCERN	Modified DISCERN	
Duration time	0.215	0.304	0.349	0.193	0.298	0.184	0.375	0.348	r
	0.324	0.159	0.103	0.377	0.082	0.290	**0.026**	**0.041**	p
Views	0.393	0.302	0.303	0.053	−0.174	−0.155	−0.131	−0.067	r
	0.064	0.161	0.160	0.812	0.350	0.404	0.483	0.721	p
Likes	0.468	0.340	0.278	0.080	−0.165	−0.222	−0.109	−0.086	r
	**0.033**	0.131	0.222	0.729	0.343	0.201	0.532	0.624	p
Comments	0.145	0.116	0.106	−0.044	−0.039	−0.164	0.005	0.038	r
	0.543	0.626	0.657	0.854	0.825	0.355	0.980	0.831	p
The days of activity	−0.388	−0.452	−0.452	−0.175	0.071	0.121	0.171	0.112	r
	0.067	**0.030**	**0.031**	0.425	0.687	0.489	0.327	0.523	p
Viewing rate	0.399	0.308	0.304	0.053	−0.186	−0.271	−0.199	−0.136	r
	0.059	0.152	0.158	0.812	0.285	0.116	0.251	0.435	p
Content analysis	0.819	0.879	0.941	0.722	0.833	0.809	0.815	0.754	r
	***p* < 0.001**	***p* < 0.001**	***p* < 0.001**	***p* < 0.001**	***p* < 0.001**	***p* < 0.001**	***p* < 0.001**	***p* < 0.001**	p
Source of video	−0.337	−0.387	−0.499	−0.730	−0.323	−0.442	−0.282	−0.312	r
	0.116	0.068	**0.015**	***p* < 0.001**	0.058	**0.008**	0.101	0.068	p

**Notes.**

r, correlation coefficient; *ρ*, Spearman’s rank correlation coefficient.

Bold values indicate statistical significance.

(Spearman correlation analysis was used to assess the relationship between variables).

## Discussion

The COVID-19 pandemic underscored the crucial role of social media in information dissemination and communication. With physical distancing measures in place, platforms like YouTube became vital for delivering updates on the virus and maintaining social connections (Bao et al., 2020). During such a sensitive period, it felt easier and quicker to get information from online platforms and communicate with people there, rather than visiting healthcare institutions. As a result, YouTube’s influence grew significantly in the post-pandemic era. As the second most visited website globally, it attracts over two billion active users each month, leveraging its free and open-access model to reach a vast audience with diverse content, including health-related information (Dean, 2025; Semrush, 2025). This widespread integration of social media into daily life has raised concerns about the quality and reliability of health-related content available online. Recognizing these concerns, healthcare professionals have increasingly sought to evaluate the credibility of such content. In particular, YouTube allows any individual to upload videos without any regulatory oversight, increasing the risk of misinformation dissemination. Healthcare-related videos, including those on dentistry and periodontology, have been critically examined by multiple professionals to assess their reliability (Hamdan et al., 2019; Aljoghaiman et al., 2024; Ince Kuka & Gursoy, 2024; Gezer et al., 2025).

First 100 videos on selected keywords regarding gingival recession were evaluated in the present study. Various studies on different topics have analyzed the first 60 to 150 using keywords (Hamdan et al., 2019; Aljoghaiman et al., 2024; Ince Kuka & Gursoy, 2024; Gezer et al., 2025). Some authors base their method on the idea that users typically engage in an information triage process when searching for health-related content online. Desai et al. (2013) noted that 95% of users view only the first three pages on YouTube. Building on these findings, in the present study 100 videos were analyzed for each period using the selected keyword. However, since the current YouTube interface does not allow filtering by a specific date range, we conducted our search in Google’s video search section, applying filters only for date and source (YouTube).

Descriptive statistics revealed that there were no significant differences between video characteristics from different time periods in the present study. The median values of duration time, number of likes and comments of videos posted in 2019 were insignificantly higher than those for videos shared in 2024, while the number of views was lower. A recent study analyzed videos on the same platform related to gingival grafts and reported an average VIQI score of 13.8 (Aljoghaiman et al., 2024). The similarity of the average VIQI score in the 2024 group of this study may be attributed to the close proximity of the analysis periods. Additionally, quality scores (GQS and VIQI) were insignificantly lower in videos posted in 2019 than those of 2024, while mean reliability score remained similar (39 ± 14, 40 ± 17, respectively), which is lower than the mean DISCERN value of Gezer et al. (49.24 ± 15.22). Similarly, Hamdan et al. (2019) found higher duration time and lower quality scores (GQS) from 2016 to 2017. Differently from the present study, they found higher number of views and fewer included videos. This difference may result from observation methodology (comparison of different time periods *versus* change over time), evaluation methodology (median values *vs* mean values, according to data distribution) and possible COVID-19 pandemic effect.

In the present study, videos were evaluated using a content analysis designed by the authors, consisting of 10 questions. Previous studies have also included evaluations such as the usefulness score (Gezer et al., 2025) and comprehensiveness index (Hamdan et al., 2019), both designed by the authors. In the present study, videos classified as useful had significantly longer durations and higher DISCERN, Modified DISCERN, VIQI, and GQS scores. Similarly, Ince Kuka & Gursoy (2024) reported that videos with higher total content scores, longer durations and greater interaction indices and GQS scores were significantly more prevalent in the more useful category, highlighting a consistent pattern across studies. On the contrary, Gezer et al. (2025) found no positive correlation between video length and usefulness score, which they explained with the tendency of higher video length but lower usefulness score of videos shared by non-professionals. Aksoy & Topsakal (2022) also found that DISCERN and GQS scores were positively affected by video content in their research on pediatric oral health instructions. Regarding source, the number of videos shared by dental professionals was higher than those of others, which was in line with studies performed by Ince Kuka & Gursoy (2024) and Gezer et al. (2025). However, this difference was only significant in 2024, which may be attributed to the increase in the sharing tendency of healthcare professionals to these platforms. Ince Kuka & Gursoy (2024) similarly highlighted the need of videos shared by healthcare professionals in their recent study on a different topic (oral hygiene).

It’s important to recognize that YouTube content is dynamic, and the quality of videos can shift over time due to changes in algorithms, content creator trends and shifting public interests. Additionally, the impact of the COVID-19 pandemic may have resulted in a greater emphasis on health-related topics, leading to higher quality or more professionally produced videos during the post-pandemic period, which could explain the rise in useful videos in our study. In line with previous studies that have shed light on our research, the importance of the video source is again highlighted (Li et al., 2020; Gezer et al., 2025). When a time difference is considered, Hamdan et al. (2019) assessed a one-year difference, and to our knowledge, this research is the first to examine the impact of the period before and after the COVID-19 pandemic on this spesific content of YouTube videos. As patients increasingly rely on digital platforms for oral health guidance, the need for credible, expert-driven content on gingival recession becomes even more apparent, reinforcing the importance of video source reliability in influencing patient perceptions and decisions.

The possible limitations of this study include analyzing only 100 videos, focusing exclusively on English-language videos and the possibility that videos outside this sample could have influenced the results.

## Conclusions

In today’s digital landscape, people increasingly turn to social media platforms to seek answers to their health-related questions. Patients experiencing issues like gingival recession—which may lead to both esthetic and functional concerns—often consult platforms like YouTube in search of guidance and reassurance. Based on the analyzed sample, videos uploaded by dental professionals demonstrated higher quality and reliability scores, highlighting the value of expert involvement in online health communication. Therefore, increasing the presence of healthcare professionals on such platforms may help enhance the availability of accurate, evidence-based content and reduce the risk of misinformation in public oral health education.

##  Supplemental Information

10.7717/peerj.19653/supp-1 Supplemental Information 1 Raw data

## Additional Information and Declarations

### Data Availability

The following information was supplied regarding data availability: The raw measurements are available in Supplementary File-1 named Raw data-Yaman (xcl file). The file shows included videos of two different time periods with description data in sheet 1 and 2. Sheet 3 comprises of all the indices used for the evaluation. Sheet 4 shows raw data of statistical analysis (agreement level of examiners, comparison based on years, content analysis, correlation between video characteristics and the indices).
